# Inhibition of TRPA1 Attenuates Doxorubicin-Induced Acute Cardiotoxicity by Suppressing Oxidative Stress, the Inflammatory Response, and Endoplasmic Reticulum Stress

**DOI:** 10.1155/2018/5179468

**Published:** 2018-02-28

**Authors:** Zhen Wang, Menglong Wang, Jianfang Liu, Jing Ye, Huimin Jiang, Yao Xu, Di Ye, Jun Wan

**Affiliations:** ^1^Department of Cardiology, Renmin Hospital of Wuhan University, Wuhan 430060, China; ^2^Cardiovascular Research Institute, Wuhan University, Wuhan 430060, China; ^3^Hubei Key Laboratory of Cardiology, Wuhan 430060, China

## Abstract

The transient receptor potential ankyrin 1 (TRPA1) channel is expressed in cardiomyocytes and involved in many cardiovascular diseases. However, the expression and function of TRPA1 in doxorubicin- (Dox-) induced acute cardiotoxicity have not been elucidated. This study aimed at investigating whether blocking the TRPA1 channel with the specific inhibitor HC-030031 (HC) attenuates Dox-induced cardiac injury. The animals were randomly divided into four groups: control, HC, Dox, and Dox + HC. Echocardiography was used to evaluate cardiac function, and the heart was removed for molecular experiments. The results showed that the expression of TRPA1 was increased in the heart after Dox treatment. Cardiac dysfunction and increased serum CK-MB and LDH levels were induced by Dox, but these effects were attenuated by HC treatment. In addition, HC mitigated Dox-induced oxidative stress, as evidenced by the decreased MDA level and increased GSH level and SOD activity in the Dox + HC group. Meanwhile, HC treatment lowered the levels of the proinflammatory cytokines IL-1*β*, IL-6, IL-17, and TNF-*α* induced by Dox. Furthermore, HC treatment mitigated endoplasmic reticulum (ER) stress and cardiomyocyte apoptosis induced by Dox. These results indicated that inhibition of TRPA1 could prevent Dox-induced cardiomyocyte apoptosis in mice by inhibiting oxidative stress, inflammation, and ER stress.

## 1. Introduction

Doxorubicin (Dox), an anthracycline anticancer drug, is one of the most preferred agents for the treatment of different malignant tumors, including leukemia, lymphomas, breast cancer, and ovarian cancer. However, its application is hampered due to a significant dose-dependent cardiotoxicity manifested by cardiomyopathy and congestive heart failure [1, 2]. A recent study reported that 21% of patients developed chemotherapy-related cardiotoxicity after Dox administration [3]. Therefore, considerable efforts have been made to identify an effective therapeutic target for mitigating Dox-induced cardiac damage.

Transient receptor potential (TRP) channels are nonselective cation channels that mediate sensory transduction and respond to various stimuli. The 28 mammalian TRP channels can be grouped into six subfamilies based on sequence homology. Among them, TRPA1 is predominantly expressed in nociceptive neurons and is also expressed at high levels in the heart, lung, skeletal muscle, skin, and vascular endothelial cells [4, 5]. It is well established that oxidative stress metabolites, such as reactive oxygen species (ROS) and specific metabolites of lipid peroxidation, are endogenous agonists of TRPA1 [6]. Takahashi et al. demonstrated that TRPA1 directly detects molecular oxygen and plays a pivotal role in maintaining oxygen homeostasis [7]. In addition, mounting evidence suggests that TRPA1 may be a key gatekeeper in detecting stimuli and regulating the inflammatory response [8, 9].

Accumulating evidence indicates the important role of TRPA1 in the pathophysiology of cardiac disease [10]. TRPA1 activators given prophylactically could reduce the infarct size in a rat model of myocardial ischemia-reperfusion injury [11]. However, the role of TRPA1 in Dox-induced cardiotoxicity is still unknown. In the present study, we clearly show that the inhibition of TRPA1 ameliorated Dox-induced cardiomyocyte apoptosis and cardiac dysfunction, which correlated with decreases in oxidative stress products, proinflammatory cytokine levels, and endoplasmic reticulum (ER) stress.

## 2. Materials and Methods

### 2.1. Animals

All procedures involving animals were conducted in compliance with the National Institutes of Health (NIH) Guide for the Care and Use of Laboratory Animals and were approved by the Ethics Committee for Animal Research of Wuhan University (Wuhan, China). Male C57BL/6J mice, aged 6–8 weeks and weighing 23–25 g, were obtained from Vital River Laboratory Animal Technology Co. Ltd. (Beijing, China). Mice were acclimatized for 7 days before assignment to their experimental groups and housed in a light-controlled room (12 h light/dark cycle) with free access to standard chow and water. The animals (*n* = 80) were randomly divided into four treatment groups of 20 mice each: control (CTRL), HC-030031 (HC), Dox, and Dox + HC. The CTRL and HC alone groups received an equivalent volume of placebo or HC orally for ten consecutive days. Dox-treated mice were injected with a single dose of Dox dissolved in normal saline (20 mg/kg i.p.) at day 5. Mice in the Dox + HC group were pretreated with HC (10 mg/kg) for 5 days by gavage and then treated for 5 additional days after the injection of Dox.

### 2.2. Echocardiography

Echocardiography was performed in anesthetized (1.5–2% isoflurane) mice using a Mylab30CV ultrasound (Biosound Esaote Inc.) equipped with a 10 MHz linear array ultrasound transducer. The left ventricle (LV) was assessed in both parasternal long-axis and short-axis views. End-systole and end-diastole were defined as the phases in which the smallest and largest areas of the LV were obtained, respectively. LV ejection fraction (EF) and LV fractional shortening (FS) were measured via LV M-mode tracing with a sweep speed of 50 mm/s at the midpapillary muscle level.

### 2.3. Biochemical Determination

Blood was collected, and the serum was separated by centrifugation. Serum concentrations of creatine kinase isoenzymes (CK-MB) and lactate dehydrogenase (LDH) in different treatment groups were measured by an automatic biochemical analyzer (ADVIA® 2400, Siemens Ltd., China).

### 2.4. Oxidative Stress Detection

At the end of the experiment, the cardiac tissues were removed and washed in ice-cold phosphate-buffered saline. The cardiac tissues (30 mg) were added to 300 *μ*l of phosphate-buffered saline, ground into homogenates, and centrifuged at 3000 rpm at 4°C for 15 min to collect the supernatant. The activities of superoxide dismutase 1 (SOD) and the content of malondialdehyde (MDA) and glutathione (GSH) were detected by commercially available kits purchased from Nanjing Jiancheng Bioengineering Institute (Nanjing, China).

### 2.5. Histological Analysis

Hearts were arrested in diastole with 10% potassium chloride solution, fixed by perfusion with 10% paraformaldehyde, and embedded in paraffin. Subsequently, heart paraffin blocks were transversely sectioned at 4-5 *μ*m, stained with hematoxylin, and eosin (H&E) for histopathology, and then visualized by light microscopy.

### 2.6. Western Blot

Protein was extracted from left ventricular tissue, and the protein concentration was assessed using a BCA protein assay kit (23,227, Thermo Fisher Scientific, Waltham, MA, USA). Protein (50 *μ*g) was separated by 10% sodium dodecyl sulfate-polyacrylamide gel electrophoresis (SDS-PAGE), transferred onto polyvinylidene fluoride membranes (IPFL00010, Millipore, Billerica, MA, USA), and incubated with different primary antibodies. The following primary antibodies were used: TRPA1 (1 : 1000 dilution, NOVUS), GAPDH (1 : 1000 dilution, Cell Signaling Technology), cleaved caspase-3 (1 : 1000 dilution, Cell Signaling Technology), Bax (1 : 1000 dilution, Cell Signaling Technology), Bcl-2 (1 : 1000 dilution, Cell Signaling Technology), Phospho-NF-*κ*B p65 (1 : 1000 dilution, Cell Signaling Technology), CHOP (1 : 1000 dilution, Cell Signaling Technology), Phospho-eIF2*α* (1 : 1000 dilution, Cell Signaling Technology), caspase-12 (1 : 1000 dilution, Cell Signaling Technology), NF-*κ*B p65 (1 : 1000 dilution, Bioworld), Nox2 (1 : 200 dilution, Santa Cruz Biotechnology), Nox4 (1 : 200 dilution, Santa Cruz Biotechnology), GRP78 (1 : 200 dilution, Santa Cruz Biotechnology), ATF-6*α* (1 : 200 dilution, Santa Cruz Biotechnology), and XBP-1 (1 : 200 dilution, Santa Cruz Biotechnology). The secondary antibody, goat anti-rabbit IgG (926–32,211; LI-COR), was incubated with the membrane for 1 h. The bands were visualized using a two-colored infrared imaging system (Odyssey; LI-COR) to quantify protein expression. The protein expression levels were normalized to GAPDH levels.

### 2.7. Real-Time Polymerase Chain Reaction Analysis

RNA was collected from LV tissue using TRIzol (15596026; Invitrogen Life Technologies, Carlsbad, CA, USA). cDNA was synthesized from 2 g of RNA from each group using oligo (DT) primers and the Transcriptor First Strand cDNA Synthesis Kit (04896866001; Roche). Quantitative analysis was conducted using a LightCycler 480 and SYBR Green Master Mix (04707516001; Roche). All details about the primers are presented in Table 1.

### 2.8. Statistical Analysis

Data are presented as the mean ± S.D. Comparisons between groups were made using analysis of variance (ANOVA), followed by Dunnett's test or Tukey's test. Differences with a *P* value less than 0.05 were considered significant.

## 3. Results

### 3.1. Dox Treatment Increases Cardiac TRPA1 Expression

**Figure 1:**
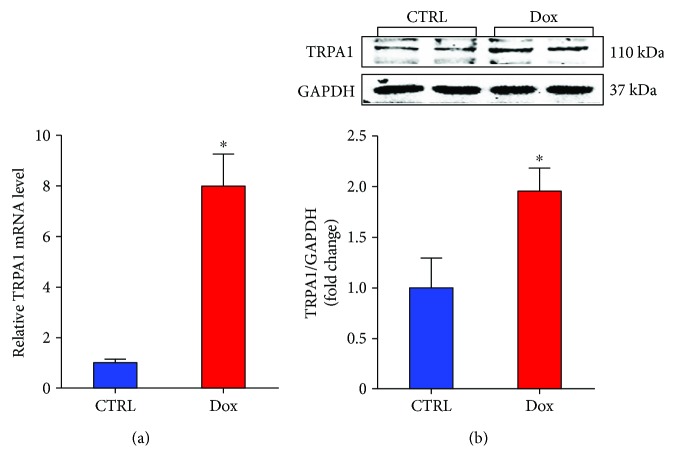
Dox treatment increases TRPA1 expression in heart tissue. (a) The relative mRNA levels of TRPA1 in the left ventricle of mice from the indicated groups. (b). Representative Western blot bands and quantitative results of protein levels of TRPA1 in Dox-induced cardiac injury. (*n* = 6). ^∗^*P* < 0.05 versus CTRL.

To investigate the potential role of TRPA1 in the development of Dox-induced myocardial lesions, we first examined the expression of TRPA1 in the heart after Dox treatment. The RT-PCR results showed that Dox treatment enhanced myocardial TRPA1 mRNA levels (Figure 1(a)). Then, western blot results showed the same trend for TRPA1 expression in the Dox-treated heart (Figure 1(b)). These results suggested that TRPA1 expression is induced by Dox treatment and that TRPA1 may be involved in Dox-induced cardiotoxicity.

### 3.2. Inhibition of TRPA1 Ameliorates Cardiac Dysfunction in Mice Treated with Dox

**Figure 2:**
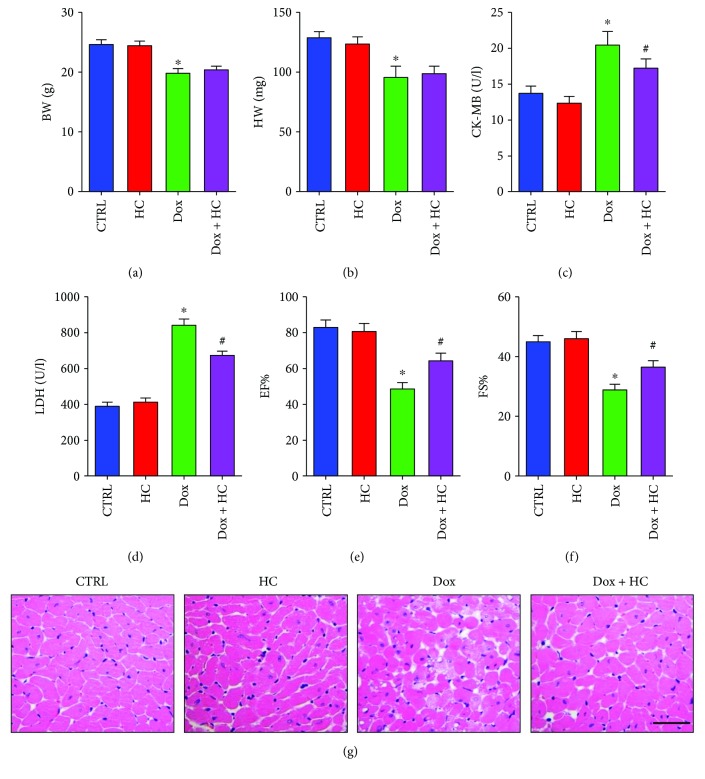
Inhibition of TRPA1 ameliorates cardiac function in mice treated with Dox. (a, b) The body weight (BW) and heart weight (HW) in different groups (*n* = 10). (c, d) The serum levels of cardiotoxicity markers, including creatine kinase isoenzymes (CK-MB) and lactate dehydrogenase (LDH) (*n* = 6). (e, f) The echocardiographic parameters in different groups (*n* = 8). (g) The pathological structure indicated by HE staining (scale bar, 50 *μ*m) (*n* = 6). ^∗^*P* < 0.05 compared with the CTRL group. ^#^*P* < 0.05 compared with the Dox group. EF: ejection fraction; FS: fractional shortening.

To explore the potential function of TRPA1 in Dox-induced cardiotoxicity, the TRPA1-specific inhibitor HC was applied for 5 days before and after Dox treatment. We first evaluated the body weight (BW) and heart weight (HW) of mice in each group. Compared to control mice, mice treated with Dox showed a decrease in BW and HW (Figures 2(a)-2(b)). However, HC treatment did not improve the decreased BW and HW induced by Dox. The expression of serum enzymes such as CK-MB and LDH, which reflect cardiac injuries, was significantly increased after the administration of Dox (Figures 2(c)-2(d)). Interestingly, the administration of HC significantly decreased the level of serum enzymes, indicating attenuated cardiotoxicity. In addition, the decreased cardiac ejection fraction (EF) and fractional shortening (FS) in the Dox group were significantly improved by HC treatment (Figures 2(e)-2(f)). Histological examination revealed increased vacuolar and myofibrillar disorganization in Dox-treated mice, and these effects were significantly ameliorated in the Dox + HC group (Figure 2(g)). The HC alone group did not show any significant changes in any of these markers compared to the control group (Figures 2(a)–2(g)).

### 3.3. Inhibition of TRPA1 Protects against Dox-Induced Oxidative Stress in Cardiac Tissue

**Figure 3:**
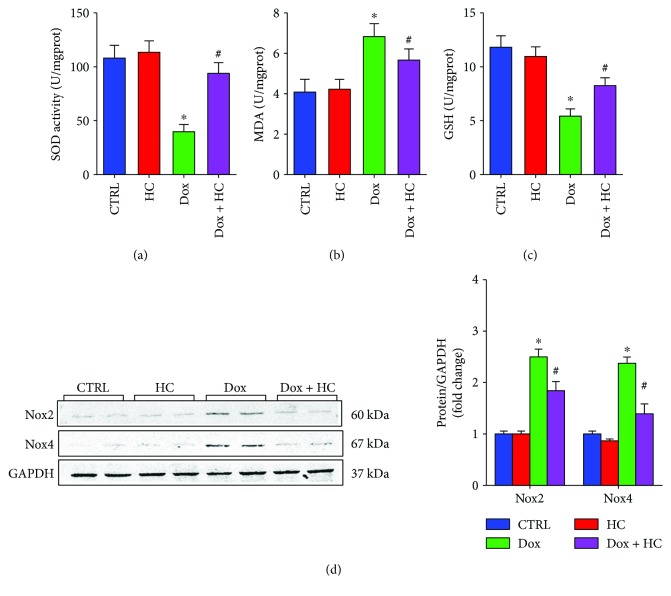
Inhibition of TRPA1 protects against Dox-induced oxidative stress. The serum levels of superoxide dismutase (SOD) (a), malondialdehyde (MDA) (b), and glutathione (GSH) (*n* = 6) (c). (d) Western blots showing the protein levels of Nox2 and Nox4 in different groups (*n* = 6). ^∗^*P* < 0.05 compared with the CTRL group, ^#^*P* < 0.05 compared with the Dox group.

Dox treatment caused a significant reduction in the activities of SOD and GSH and an increase in the levels of MDA compared with the control group (Figures 3(a)–3(c)). However, HC treatment significantly decreased MDA levels and restored SOD activity and GSH antioxidant levels compared with the Dox-treated mice (Figures 3(a)–3(c)). Furthermore, the expression of Nox2 and Nox4, which are important generators of ROS, was lower in the Dox + HC group compared with the Dox group (Figure 3(d)). These findings indicate that HC treatment decreases the cardiac oxidative stress induced by Dox.

### 3.4. Inhibition of TRPA1 Reduces Dox-Induced Inflammation in Cardiac Tissue

**Figure 4:**
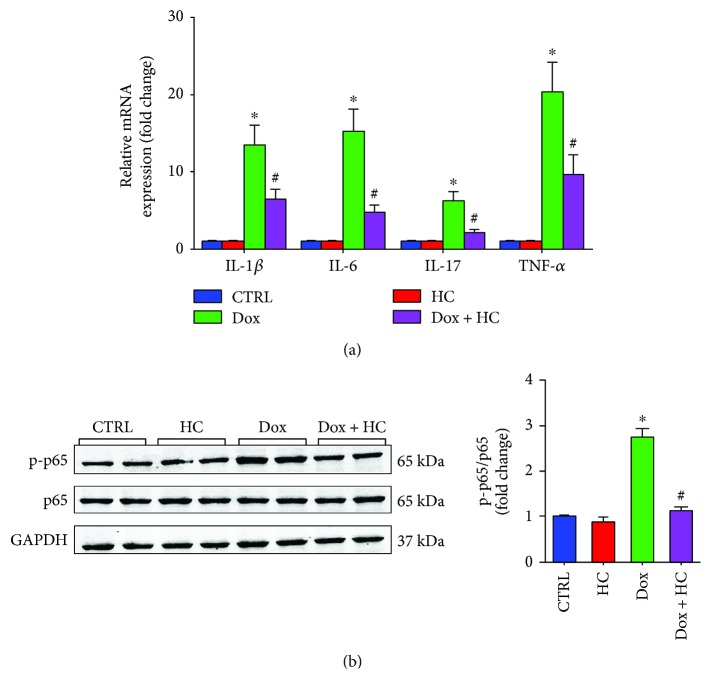
Inhibition of TRPA1 protects against Dox-induced inflammatory in cardiac tissue. (a) The mRNA expression of inflammatory cytokines, including Il-1*β*, IL-6, IL-17, and TNF-*α*, in different groups (*n* = 6). (b) Western blot analysis of p65 and p-p65 in different groups (*n* = 6). ^∗^*P* < 0.05 compared with the CTRL group, ^#^*P* < 0.05 compared with the Dox group.

As shown in Figure 4, the expression in the heart of proinflammatory cytokines, including IL-1*β*, IL-6, IL-17, and TNF-*α*, was significantly increased by Dox (Figure 4(a)). Conversely, significant reductions in IL-1*β*, IL-6, IL-17, and TNF-*α* were observed in the Dox + HC group compared with the Dox group (Figure 4(a)). In addition, the inhibitory effects of HC on inflammation were further confirmed by western blot results showing that HC reduced NF-*κ*B signaling (Figure 4(b)). These results demonstrate that HC protects against heart injury by inhibiting inflammatory responses.

### 3.5. Inhibition of TRPA1 Attenuates Dox-Induced ER Stress

**Figure 5:**
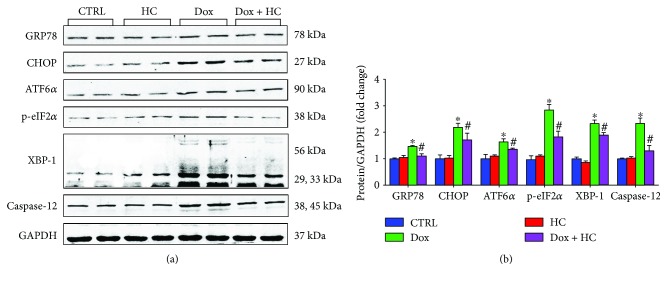
Inhibition of TRPA1 attenuates Dox-induced ER stress. Representative western blots (a) and quantitative results (b) showing the expression of glucose-regulated protein 78 (GRP78), C/EBP homologous protein (CHOP), activating transcription factor 6 (ATF6), eukaryotic translation initiation factor 2 (p-eIF2), X-box binding protein 1 (XBP-1), and caspase-12 in different groups (*n* = 6). ^∗^*P* < 0.05 compared with the CTRL group, ^#^*P* < 0.05 compared with the Dox group.

Emerging evidence suggests that ER stress plays a crucial role in Dox-induced cardiotoxicity [12, 13]. Thus, we investigated whether the cardioprotective effects of HC against Dox-induced cardiotoxicity are associated with decreased ER stress. The results showed that HC treatment suppressed the expression of glucose-regulated protein 78 (GRP78), an important marker indicating the severity of ER stress. In addition, we found that Dox induction increased the levels of C/EBP homologous protein (CHOP) and cleaved caspase-12, important mediators of ER stress-induced apoptosis, and this induction was attenuated by HC treatment. Furthermore, the activation of ER stress signaling pathways was inhibited by HC treatment, as evidenced by the decreased expression of activating transcription factor 6 (ATF6), eukaryotic translation initiation factor 2*α* (eIF2*α*), and X-box binding protein 1 (XBP-1) in the Dox + HC group (Figure 5). These results indicate that HC treatment attenuates the ER stress induced by Dox.

### 3.6. Inhibition of TRPA1 Attenuates Dox-Induced Cardiomyocyte Apoptosis

**Figure 6:**
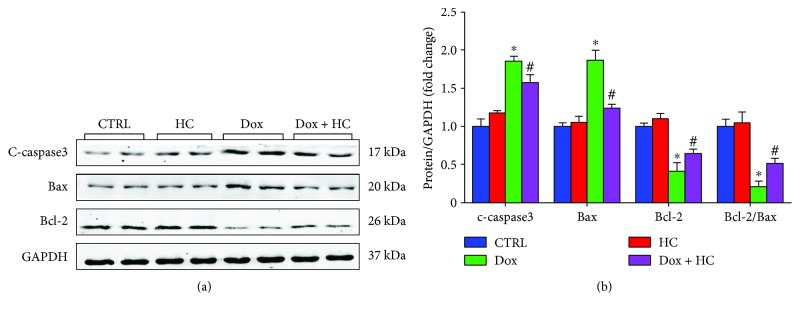
Inhibition of TRPA1 attenuates Dox-induced cardiomyocyte apoptosis. Representative western blots (a) and quantitative results (b) showing the expression of c-caspase-3, Bax, and Bcl-2 in different groups (*n* = 6). ^∗^*P* < 0.05 compared with the CTRL group, ^#^*P* < 0.05 compared with the Dox group.

It is well known that apoptosis is involved in Dox-induced cardiotoxicity [14, 15]. We evaluated the severity of apoptosis and identified the potential signaling pathways related to apoptosis in the heart. The levels of Bax and cleaved caspase-3 in myocardial tissue were upregulated in the Dox group compared with the control group (Figure 6). By contrast, the expression level of Bcl-2 was significantly lower in the Dox group than in the control group. The HC alone group did not show any significant changes in any of these markers compared to the control group (Figure 6). However, HC treatment significantly attenuated the increased Bax and cleaved caspase-3 levels and improved the expression of Bcl-2 after Dox treatment. These findings demonstrate that HC can decrease Dox-induced cardiomyocyte apoptosis.

## 4. Discussion

Cardiotoxicity is induced by a single intraperitoneal injection of Dox (20 mg/kg) in mice, which triggers the development of cardiac dysfunction and congestive heart failure [16, 17]. The present study demonstrated the potential role of TRPA1 in Dox-induced cardiotoxicity and elucidated the potential underlying molecular mechanisms. First, we observed that the expression level of TRPA1 was upregulated in the heart after Dox treatment. Moreover, we demonstrated that inhibition of TRPA1 with the specific inhibitor HC ameliorated Dox-induced cardiac injuries, as evidenced by attenuated heart dysfunction, structural damage, oxidative stress, inflammatory response, and ER stress. More importantly, Dox-induced cardiomyocyte apoptosis was attenuated by HC treatment. These findings imply that the inhibition of TRPA1 could effectively attenuate the progression of Dox-induced cardiotoxicity.

Redox homeostasis, which depends on the fine balance between enzymatic cascades, serves a pivotal role in adaptive responses under stress conditions. However, uncontrolled accumulation of reactive oxygen species (ROS), a state known as oxidative stress, occurs during tissue damage and impaired cell function [18, 19]. It is well established that TRPA1 acts as a polymodal nociceptor and molecular integrator of cellular stressors, including ROS and reactive nitrogen species (RNS) [20]. Genetic deletion of TRPA1, or the blockade of its activation with a selective antagonist, abrogated trigeminal neuropathic pain and oxidative stress [21].

In the present study, the levels of myocardium biomarkers, including lipid peroxidation products (MDA) and antioxidant enzymes (SOD and GSH), were used to estimate oxidative stress. The administration of Dox significantly increased MDA levels, reduced the activity of SOD, and reduced GSH content in the heart. Interestingly, HC treatment reduced the extent of Dox-induced oxidative stress by increasing SOD activity and MDA levels and decreasing GSH levels. Furthermore, previous studies reported that Dox could induce the production of ROS via activation of the NADPH oxidase pathway [22]. Zhao et al. found that Nox2 deficiency protected mice against cardiac injury and apoptosis after Dox treatment [23]. Consistent with these studies, we found that the expression of Nox2 and Nox4, the pivotal NADPH oxidase subunit, was upregulated after Dox administration. However, treatment with HC inhibited the oxidative stress, possibly by downregulating the expression of Nox2 and Nox4 in the heart.

The inflammatory process is absolutely essential for defense privilege that intended to eliminate or neutralize invading pathogens, clear damaged tissues, and promote their repair, but the termination of the response is of equal importance. Failure to control inflammation can lead to immunopathology, such as systemic inflammation leading to organ dysfunction and death. In previous research, TRPA1 emerged as a key regulator of sensory neuropeptide release and acute neurogenic inflammation. However, there is accumulating evidence for a link between TRPA1 and immunoinflammatory processes. In the cornea following a chemical injury, the absence of TRPA1 or TRPA1 antagonist treatment suppressed inflammation and fibrosis by decreasing levels of IL-6, TGF-*β*1, and vascular endothelial growth factor [24]. Similarly, loss of TRPA1 restrained neutrophil infiltration and proinflammatory cytokines, mainly IL-1*β*, produced by monosodium urate. These reports highlight the potential for anti-inflammatory signaling via alternative mechanisms targeting TRPA1 [25]. Besides its direct deleterious effect, Dox can also induce inflammatory responses via enhanced expression and release of proinflammatory cytokines [26, 27]. It has been demonstrated that Dox treatment induces the release of proinflammatory cytokines, such as TNF-*α*, via the activation of NF-*κ*B in the heart [28]. Studies demonstrated that the inhibition of TRPA1 results in a relevant reduction of the proinflammatory cytokines IL-1*β* and TNF-*α* in cystic fibrosis patients [29]. Similarly, our results indicated that Dox treatment provokes a series of inflammatory responses and increases the expression levels of inflammatory cytokines, which lead to the deterioration of myocardial function. Inhibition of TRPA1 significantly reduced the expression of proinflammatory cytokines, such as IL-1*β*, IL-6, IL-17, and TNF-*α* and suppressed the expression of NF-*κ*B. This study indicates that the anti-inflammatory consequences of TRPA1 inhibition may partly contribute to the potential cardioprotective effect against Dox-induced cardiotoxicity.

To further investigate the potential mechanisms behind TRPA1-mediated Dox-induced cardiotoxicity in the heart, we examined the level of ER stress that plays a pivotal role in the development of heart failure [30, 31]. Consistent with previous reports, ER stress-related proteins were enhanced in Dox-treated mice. Many studies have demonstrated that Dox promotes the endoplasmic reticulum-initiated apoptotic response by activating the expression of proapoptotic factors and inhibiting the expression of antiapoptotic factors [32]. As a specific proapoptotic pathway, ER stress can activate the CHOP and caspase-12 pathways and thereby mediate apoptosis [33]. In our study, HC treatment attenuated the expression of CHOP and caspase-12, leading to decreased myocardial apoptosis and ameliorated cardiac dysfunction. Furthermore, many studies have demonstrated that CHOP can also directly regulate apoptosis factors such as Bax, Bcl-2, and cleaved caspase-3, which are key determinants of cell death [34]. Our data also support this hypothesis, since HC treatment significantly increased the expression of Bcl-2 and decreased the expression of Bax and cleaved caspase-3.

In conclusion, our study indicates that the inhibition of TRPA1 could protect the heart from Dox-induced cardiomyocyte apoptosis and cardiac dysfunction by inhibiting oxidative stress, inflammatory responses, and ER stress. These findings suggest that TRPA1 could be a potential therapeutic target for the treatment of cardiotoxicity caused by Dox.

## Figures and Tables

**Table 1:** Primers for quantitative polymerase chain reaction.

Gene	Forward primer (5′-3′)	Reverse primer (5′-3′)
TRPA1	GTCCAGGGCGTTGTCTATCG	CGTGATGCAGAGGACAGAGAT
IL-1*β*	GGGCCTCAAAGGAAAGAATC	TACCAGTTGGGGAACTCTGC
IL-6	CCAAGAGGTGAGTGCTTCCC	CTGTTGTTCAGACTCTCTCCCT
IL-17	TTTAACTCCCTTGGCGCAAAA	CTTTCCCTCCGCATTGACAC
TNF-*α*	GACGTGGAACTGGCAGAAGAG	TTGGTGGTTTGTGAGTGTGAG
GAPDH	AACTTTGGCATTGTGGAAGG	CACATTGGGGGTAGGAACAC

## References

[1] Vejpongsa P., Yeh E. T. H. (2014). Prevention of anthracycline-induced cardiotoxicity: challenges and opportunities. *Journal of the American College of Cardiology*.

[2] Fernandez-Ruiz I. (2016). Cardioprotection: cardiotoxicity of anticancer therapy. *Nature Reviews Cardiology*.

[3] Sawaya H., Sebag I. A., Plana J. C. (2011). Early detection and prediction of cardiotoxicity in chemotherapy-treated patients. *The American Journal of Cardiology*.

[4] Nilius B., Appendino G., Owsianik G. (2012). The transient receptor potential channel TRPA1: from gene to pathophysiology. *Pflügers Archiv*.

[5] Andrei S. R., Sinharoy P., Bratz I. N., Damron D. S. (2016). TRPA1 is functionally co-expressed with TRPV1 in cardiac muscle: co-localization at z-discs, costameres and intercalated discs. *Channels*.

[6] Andersson D. A., Gentry C., Mossand S., Bevan S. (2008). Transient receptor potential A1 is a sensory receptor for multiple products of oxidative stress. *The Journal of Neuroscience*.

[7] Takahashi N., Kuwaki T., Kiyonaka S. (2011). TRPA1 underlies a sensing mechanism for O_2_. *Nature Chemical Biology*.

[8] Bautista D. M., Jordt S. E., Nikai T. (2006). TRPA1 mediates the inflammatory actions of environmental irritants and proalgesic agents. *Cell*.

[9] Bautista D. M., Pellegrino M., Tsunozaki M. (2013). TRPA1: a gatekeeper for inflammation. *Annual Review of Physiology*.

[10] Bodkin J. V., Brain S. D. (2011). Transient receptor potential ankyrin 1: emerging pharmacology and indications for cardiovascular biology. *Acta Physiologica*.

[11] Lu Y., Piplani H., McAllister S. L., Hurt C. M., Gross E. R. (2016). Transient receptor potential ankyrin 1 activation within the cardiac myocyte limits ischemia–reperfusion injury in rodents. *Anesthesiology*.

[12] Fu H. Y., Sanada S., Matsuzaki T. (2016). Chemical endoplasmic reticulum chaperone alleviates doxorubicin-induced cardiac dysfunction. *Circulation Research*.

[13] Chen R. C., Sun G. B., Ye J. X., Wang J., Zhang M. D., Sun X. B. (2017). Salvianolic acid B attenuates doxorubicin-induced ER stress by inhibiting TRPC3 and TRPC6 mediated Ca^2+^ overload in rat cardiomyocytes. *Toxicology Letters*.

[14] Wang L., Zhang T.-P., Zhang Y. (2016). Protection against doxorubicin-induced myocardial dysfunction in mice by cardiac-specific expression of carboxyl terminus of hsp70-interacting protein. *Scientific Reports*.

[15] Rehman M. U., Tahir M., Khan A. Q. (2014). D-limonene suppresses doxorubicin-induced oxidative stress and inflammation via repression of COX-2, iNOS, and NF*κ*B in kidneys of Wistar rats. *Experimental Biology and Medicine.*.

[16] Li K., Sung R. Y., Huang W. Z. (2006). Thrombopoietin protects against in vitro and in vivo cardiotoxicity induced by doxorubicin. *Circulation*.

[17] Kobayashi M., Usui F., Karasawa T. (2016). NLRP3 deficiency reduces macrophage interleukin-10 production and enhances the susceptibility to doxorubicin-induced cardiotoxicity. *Scientific Reports*.

[18] Schieber M., Chandel N. S. (2014). ROS function in redox signaling and oxidative stress. *Current Biology*.

[19] Halliwell B. (2012). Free radicals and antioxidants: updating a personal view. *Nutrition Reviews*.

[20] Takahashi N., Mori Y. (2011). TRP channels as sensors and signal integrators of redox status changes. *Frontiers in Pharmacology*.

[21] Trevisan G., Benemei S., Materazzi S. (2016). TRPA1 mediates trigeminal neuropathic pain in mice downstream of monocytes/macrophages and oxidative stress. *Brain*.

[22] Wojnowski L., Kulle B., Schirmer M. (2005). NAD(P)H oxidase and multidrug resistance protein genetic polymorphisms are associated with doxorubicin-induced cardiotoxicity. *Circulation*.

[23] Zhao Y., McLaughlin D., Robinson E. (2010). Nox2 NADPH oxidase promotes pathologic cardiac remodeling associated with doxorubicin chemotherapy. *Cancer Research*.

[24] Okada Y., Shirai K., Reinach P. S. (2014). TRPA1 is required for TGF-*β* signaling and its loss blocks inflammatory fibrosis in mouse corneal stroma. *Laboratory Investigation*.

[25] Trevisan G., Hoffmeister C., Rossato M. F. (2014). TRPA1 receptor stimulation by hydrogen peroxide is critical to trigger hyperalgesia and inflammation in a model of acute gout. *Free Radical Biology & Medicine*.

[26] Pecoraro M., Del Pizzo M., Marzocco S. (2016). Inflammatory mediators in a short-time mouse model of doxorubicin-induced cardiotoxicity. *Toxicology and Applied Pharmacology*.

[27] Wang Z. Q., Chen M. T., Zhang R., Zhang Y., Li W., Li Y. G. (2016). Docosahexaenoic acid attenuates doxorubicin-induced cytotoxicity and inflammation by suppressing NF-*κ*B/iNOS/NO signaling pathway activation in H9C2 cardiac cells. *Journal of Cardiovascular Pharmacology*.

[28] Abd E. T., Mohamed R. H., Pasha H. F., Abdel-Aziz H. R. (2012). Catechin protects against oxidative stress and inflammatory-mediated cardiotoxicity in adriamycin-treated rats. *Clinical and Experimental Medicine*.

[29] Prandini P., De Logu F., Fusi C. (2016). Transient receptor potential ankyrin 1 channels modulate inflammatory response in respiratory cells from patients with cystic fibrosis. *American Journal of Respiratory Cell and Molecular Biology*.

[30] Minamino T., Komuro I., Kitakaze M. (2010). Endoplasmic reticulum stress as a therapeutic target in cardiovascular disease. *Circulation Research*.

[31] Minamino T., Kitakaze M. (2010). ER stress in cardiovascular disease. *Journal of Molecular and Cellular Cardiology*.

[32] Chua C. C., Gao J., Ho Y. S. (2009). Over-expression of a modified bifunctional apoptosis regulator protects against cardiac injury and doxorubicin-induced cardiotoxicity in transgenic mice. *Cardiovascular Research*.

[33] Tabas I., Ron D. (2011). Integrating the mechanisms of apoptosis induced by endoplasmic reticulum stress. *Nature Cell Biology*.

[34] Fu H. Y., Okada K.-i., Liao Y. (2010). Ablation of C/EBP homologous protein attenuates endoplasmic reticulum–mediated apoptosis and cardiac dysfunction induced by pressure overload. *Circulation*.

